# The intersection between circulatory microRNAs and biomarkers of neurodegeneration

**DOI:** 10.1002/alz70861_108906

**Published:** 2025-12-23

**Authors:** Amber Yaqub, Rima Mustafa, Mohammad Arfan Ikram, Mohsen Ghanbari

**Affiliations:** ^1^ Department of Epidemiology, Erasmus University Medical Center, Rotterdam, Zuid Holland Netherlands; ^2^ Imperial College London, London UK; ^3^ Department of Epidemiology, Erasmus University Medical Center, Rotterdam Netherlands; ^4^ Department of Epidemiology, Erasmus Medical Centre, Rotterdam Netherlands

## Abstract

**Background:**

MicroRNAs (miRNAs) are small non‐coding RNAs with a vast array of biological functions, including the regulation of gene expression. It remains to be determined whether circulatory miRNAs are associated with markers of neurodegeneration in plasma (neurofilament light chain (NfL), total tau (t‐tau) and amyloid beta (Aβ)), before the clinical onset of dementia.

**Method:**

We included 1959 dementia‐free participants of the population‐based Rotterdam Study (mean age 70.5 years, 56.8% women), who visited the research center for blood sampling between 2002‐2005. Plasma levels of 591 well‐expressed circulatory miRNAs were measured by the HTG EdgeSeq Whole Transcriptome Assay, while t‐tau, NfL, Aβ‐40 and Aβ‐42 were analysed using the Simoa NF‐light® and N3PA assays. We used linear regression models to determine associations between circulatory miRNAs and markers of neurodegeneration, while adjusting for potential confounders. To account for the high‐dimensional structure of miRNAs, we repeated the analysis using elastic net regularization models. Finally, we performed a post‐hoc in‐silico analysis to study the common miRNAs between all four biomarkers, their potential target genes and their link to dementia.

**Result:**

We found 58 miRNAs significantly associated with t‐tau, 96 miRNAs with NfL, 158 miRNAs with Aβ‐40, and 83 miRNAs with Aβ‐42 (all with false discovery rate (FDR)‐adjusted *p* ‐value ≤ 0.05). Notably, twelve miRNAs (miR‐3141, miR‐7107‐5p, miR‐146a‐5p, miR‐6887‐5p, miR‐221‐3p, miR‐4681, miR‐6810‐3p, miR‐6821‐5p, miR‐654‐5p, miR‐4486, miR‐4478, miR‐326) were shared among all four neurodegeneration biomarkers. Subsequent in‐silico analysis showed that many of these miRNAs are expressed across various brain regions, where they have important putative target genes (e.g., *SORT1, TSPAN14, ADAM17, KLF16, WDR12*). A pathway analysis highlighted the Notch signalling cascade, with possible implications for the amyloid precursor protein (*APP*).

**Conclusion:**

This population‐based study revealed many circulatory miRNAs associated with biomarkers of neurodegeneration, including 12 miRNAs that were common to all, potentially offering valuable insights into regulatory pathways underlying dementia. These miRNAs could be valuable for monitoring dementia and developing effective diagnostic or therapeutic strategies.

